# Hypoglycemic risk exposures in relation to low serum glucose values in ambulatory patients

**DOI:** 10.1097/MD.0000000000018679

**Published:** 2020-01-10

**Authors:** Mohammed S. Abusamaan, Mark A. Marzinke, Aditya Ashok, Karen Carroll, Kyrstin Lane, Rebecca Jeun, Kendall F. Moseley, Kathryn A. Carson, Nestoras N. Mathioudakis

**Affiliations:** a Division of Endocrinology, Diabetes, & Metabolism; b Department of Pathology, Johns Hopkins University School of Medicine; c Department of Epidemiology, Johns Hopkins Bloomberg School of Public Health, Baltimore, MD.

**Keywords:** ambulatory, critical action value, glucose, hypoglycemia, laboratory, risk factors

## Abstract

This study aimed to correlate hypoglycemic risk exposures (HREs) with low blood glucose value (BGV) in ambulatory patients to inform selection of a glucose critical action value (CAV).

This was a retrospective study of ambulatory patients with at least 1 serum glucose ≤70 mg/dL obtained at 2 laboratories within the Johns Hopkins Health System over 3.8 years. Multivariable logistic regression was used to evaluate association of BGV cut-offs of <60, <54, <50, and <45 mg/dL with HREs. HREs were classified as “high hypoglycemic risk” (HHR), “moderate hypoglycemic risk” (MHR), “low hypoglycemic risk” (LHR), and “no hypoglycemic risk” (NHR).

A total of 5404 patient samples of BG ≤70 mg/dL were analyzed, of which 30.3%, 23.2%, 28.5%, 18.0% occurred in NHR, LHR, MHR, and HHR groups, respectively. An inverse relationship was noted between BGV cut-offs and HHR, but no association was observed for LHR or MHR. After adjusting for age, sex, and race, there was an inverse association between BG thresholds and the odds of HHR. For classification of HHR, BGV cut-offs of <60, <54, <50, and <45 mg/dL correctly classified 71.2%, 69.8%, 68.8%, and 67.2% of BG samples, achieved false-positive rates of 13.6%, 4.7%, 1.7%, and 0.5% and positive likelihood ratios of 3.3, 6.0, 11.2, and 23.4, respectively.

Nearly 70% of low BGVs occurred in patients with at least 1 HRE, but only ∼20% occurred in HHR patients. Given their high positive likelihood ratios, BGVs <54 or <50 mg/dL are reasonable candidates for CAVs that would allow sufficient clinician response time while minimizing false-positive alerts.

## 1 Introduction

A low blood glucose value (BGV) is a commonly encountered laboratory finding among general ambulatory patients. A diagnosis of clinically significant hypoglycemia requires assessment of a patient's signs and symptoms of low blood glucose (BG) in conjunction with labs indicating actual hypoglycemia. As such, it is unclear what action should be taken in response to a BGV below the lower limit of normal for a given laboratory assay in resulted ambulatory labs when, presumably, the patient has already left the testing site. In 2016, the International Hypoglycemia Study Group defined a BG <54 mg/dL to reflect clinically significant hypoglycemia, and a BG ≤70 mg/dL as the level at which clinicians should be alerted to the potential for symptomatic hypoglycemia.^[1]^ There is no absolute BGV that defines severe hypoglycemia, but this is generally considered to be a level at which severe cognitive impairment occurs prompting the need for third-party assistance for recovery.^[2]^

A challenge for clinical laboratories and health systems is identifying a BGV in a general population that defines a critical action value (CAV), which “represents a pathophysiological state at such variance with normal as to be life-threatening unless something is done promptly, and for which some corrective action could be taken.”^[3]^ From a patient safety perspective, this is difficult to define for several reasons. First, BGVs reflective of “life-threatening risk” would be expected to differ among diabetic and nondiabetic patients, as the former would have a higher likelihood of exposure to antihyperglycemic medications that could lead to rapid decline in BGV in the absence of intervention. Second, CAVs for glucose are not reported to the patient, but rather the provider; in an outpatient or outreach setting, there may be a delay between provider notification and patient communication. Considering that glucose can fall quickly without corrective action, this lag time needs to be factored into the selection of a glucose CAV. Finally, since patients with hypoglycemic awareness would likely self-treat if symptomatic even before being notified by a clinician of a low BGV, the clinical utility of contacting all patients with a low BGV is uncertain. Due to these aforementioned factors, the threshold at which a glucose CAV could have maximum impact is unknown, and may largely be patient-cohort-dependent.

This uncertainty is illustrated by varying CAV criteria in different patient populations at 2 academic hospitals within our health system. At the Johns Hopkins Hospital (JHH), the BG CAV for inpatients/emergency department (ED) patients and general ambulatory patients is <60 mg/dL and <50 mg/dL, respectively. At Johns Hopkins Bayview Medical Center (JHBMC), the BG CAV for outpatients is <50 mg/dL; for inpatients, the CAV criteria based on the patient's hospital length of stay: for patients admitted <7 days versus ≥ 7 days, the BG CAVs are <45 mg/dL and <50 mg/dL, respectively.

The objective of this study was to evaluate the presence of clinically significant hypoglycemic risk factors at different low BGVs in a general ambulatory patient population to better understand the implications of implementing different BG CAVs by a clinical laboratory. We hypothesized that a BGV of <54 mg/dL, which aligns with the current definition of clinically significant hypoglycemia, would capture patients at greatest risk of severe hypoglycemia due to exposure to high-risk conditions (eg, insulin and insulin secretagogue use) while minimizing false-positives (ie, patients with no hypoglycemic risk [NHR] factors or exposure only to conditions or medications expected to pose low-risk of severe hypoglycemia).

## 2 Methods

### 2.1 Patient population and design

This was a retrospective study using electronic medical records (EMR) of ambulatory patients who had a serum glucose evaluated at the clinical laboratories of the JHH and JHBMC, both tertiary care academic medical centers located in Baltimore, Maryland, between April 1, 2013 and January 31, 2017. This study was approved by the Johns Hopkins Institutional Review Board. All data were extracted from our EMR (EpicCare) and were deidentified.

Patients were eligible for inclusion if they had at least 1 serum glucose in the hypoglycemic range of ≤70 mg/dL during the study period. Point-of-care capillary glucose, and whole BG samples were excluded. Serum glucose levels were measured using the hexokinase method on the Roche cobas analyzer (glucose HK Gen.3; Roche Diagnostics, Indianapolis, IN) at JHH Core Laboratories and the hexokinase method on the Siemens Vista analyzer (Siemens Dimension Vista, Siemens AG, Erlangen, Germany) at JHBMC Core Laboratories. Serum glucose results from inpatient and ED patients were excluded. For patients who had repeated hypoglycemic readings on the same calendar day, the nadir BGV on that day was used as the index BGV in all analyses.

### 2.2 Exposures: hypoglycemic risk factors

We relied on clinical experience and review of the scientific literature to identify hypoglycemic risk exposures (HREs) for diabetic and nondiabetic populations in the ambulatory setting. Our goal was to be as comprehensive as possible to maximize the sensitivity of detecting any clinically relevant hypoglycemic risk. A total of 25 hypoglycemic risk factors were included. Diagnoses were extracted from the EMR using a combination of relevant International Classification of Diseases (ICD)-10 codes from the problem list, patient encounter, past medical history and laboratory data available on or before the index low BGV. All medications were extracted from the medication history section of the EMR. Active medication use was considered to be a prescription for the relevant drug class entered on or before the index hypoglycemic episode. Prescriptions written by clinicians outside our EMR system were not captured.

Based on clinical experience and literature review with a particular focus on the degree of expected hypoglycemia and prevalence of reported hypoglycemia associated with each condition, we classified HREs as “high hypoglycemic risk” (HHR), “moderate hypoglycemic risk” (MHR), and “low hypoglycemic risk” (LHR). The category of NHR was applied to patients for whom we could not identify a single hypoglycemic risk factor in relation to the index BGV. Table 1 defines the criteria used to identify each HRE and their classification into hypoglycemic risk categories. HHR factors were type 1 diabetes mellitus (T1DM),^[4–7]^ diabetes mellitus secondary to cystic fibrosis (CF),^[8]^ post-pancreatectomy diabetes,^[9]^ established hypoglycemic disorders,^[10]^ insulinoma,^[4]^ adrenal insufficiency,^[4,11]^ insulin use,^[4,6,7]^ and insulin secretagogue use (ie, sulfonylurea, meglitinides).^[4,7]^ MHR factors were type 2 diabetes mellitus (T2DM),^[5,9]^ congestive heart failure (CHF),^[4,12–14]^ acute kidney injury (AKI),^[15]^ end-stage renal disease (ESRD),^[16–18]^ hepatic failure,^[4,11,19]^ and use of any low-risk antihyperglycemic medications (metformin, dipeptidyl-peptidase-4 inhibitors, glucagon-like-1 receptor agonists, thiazolidinediones, and alpha glucosidase inhibitors).^[20]^ LHR factors were chronic liver disease (CLD),^[11,19]^ alcohol abuse,^[4,19,21,22]^ opioid use,^[23,24]^ human immunodeficiency virus (HIV),^[25]^ malignancy,^[26]^ malnutrition and eating disorders,^[11]^ post-bariatric or gastrointestinal surgery,^[27–30]^ intestinal malabsorption,^[31]^ indomethacin use,^[32]^ lithium use,^[32]^ and fluoroquinolone use.^[32]^ If a patient had multiple hypoglycemic risk factors across different hypoglycemic risk categories, their hypoglycemic risk was category was designated according to the condition in the highest risk category. For example, a patient with a diagnosis of T1DM (HHR condition) and ESRD (MHR condition) was classified as HHR. We made the assumption that hypoglycemic risk category would be inversely associated with BG concentration and directly associated with hypoglycemic signs and symptoms.

**Table 1:**
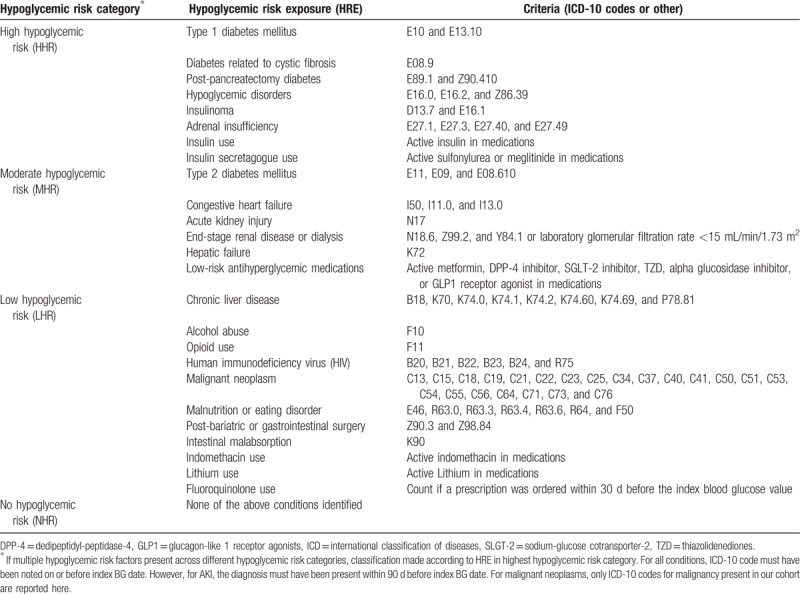
Classification of hypoglycemic risk exposures.

If ICD-10 codes for T1DM and T2DM were present concurrently for the same patient, we designated the patient as having T1DM if a prescription for insulin was active in the absence of any other glucose-lowering medications; otherwise, the patient was designated as having T2DM. AKI was defined as present if the diagnosis was noted within 90 days before the index hypoglycemic episode.^[15]^ In addition to ICD-10 codes, ESRD was diagnosed using the estimated glomerular filtration rate (eGFR) value occurring in the shortest interval before the date of the index BGV, and was defined by a eGFR value of <15 mL/min/1.73 m^2^.^[33]^ Filtration rates were determined using the modification of diet in renal disease equation.^[34]^ Since antibiotics are often prescribed for short duration, fluoroquinolones were considered to be active if a prescription was ordered within 30-days before the index BGV.

### 2.3 Outcome: BG

To evaluate the association between hypoglycemic risk categories (exposure), we selected 4 BG thresholds in the hypoglycemic range as the outcome variables in this study: 60 mg/dL, 54 mg/dL, 50 mg/dL, and 45 mg/dL. The rationale for selection of these cut-offs was as follows:

(1) since many healthy adults frequently have BGVs at or above 60 mg/dL, this was selected as the least conservative threshold^[4]^;
(2) 54 mg/dL was selected to align with consensus guidelines as the definition for clinically significant hypoglycemia^[1]^;
(3) 50 mg/dL was selected because this is the BG CAV threshold currently in place for ambulatory patients at our institutions, and
(4) 45 mg/dL was selected as the most conservative threshold to reflect “severe hypoglycemia.”

For each outcome variable, a case was defined as a BGV below the threshold and a control was defined as a BGV at or above the cut-point and less than or equal to 70 mg/dL. For example, for BG threshold of 60 mg/dL, a case was defined as a BG <60 mg/dL and a control 60 to 70 mg/dL.

### 2.4 Statistical analysis

Descriptive statistics were used to summarize the patient population. For continuous measures, normality of data was assessed using histograms and tests of skewness and kurtosis. As all continuous variables were non-normally distributed, medians and interquartile ranges (IQR) are reported. For categorical variables, counts and frequencies are provided. For comparison of continuous variables across groups, Wilcoxon rank sum test was used.

Simple logistic regression was used to explore the association between the 4 BG cut-offs as the dependent outcome variables and the 3 hypoglycemic risk categories as the independent exposure variables. Multivariable logistic regression was then used to adjust for age, sex, and race, which were all significantly associated with BG outcomes on univariate analyses. In all analyses, the reference group for hypoglycemic risk categories was NHR. Because hypoglycemic episodes occurring in the same patient are not independent events, robust standard error estimates were determined using clustering analysis per unique patients.

To evaluate how the different hypoglycemic BG thresholds performed in classifying hypoglycemic risk categories, we treated the hypoglycemic risk category as the “true disease state” and the BGV as the diagnostic test to create 2-by-2 tables for calculation of the test characteristics (sensitivity, specificity, positive and negative likelihood ratios, false-positive, false-negative rates, and correct classification rate). For each “disease state,” the hypoglycemic risk categories (HHR, MHR, and LHR) were considered positive and the NHR was considered negative. For example, for evaluation of a BG <60 mg/dL in detecting HHR, a BG <60 mg/dL was considered an abnormal test result and 60 to 70 mg/dL was considered normal; HHR was considered positive and NHR was considered negative as a disease state. Statistical analyses were performed using Stata Statistical Software: Release 14 (College Station, TX). *P* < .05 was considered statistically significant.

## 3 Results

**Figure 1:**
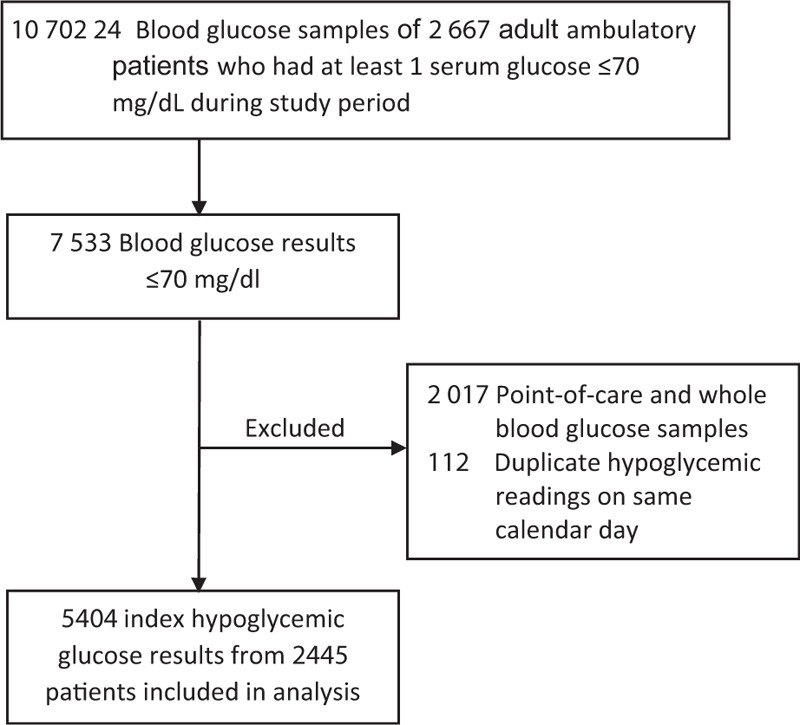
Study flowchart.

Table 2 summarizes the study population characteristics. A total of 5404 index hypoglycemic BG results ≤70 mg/dL from 2445 unique patients were included in the analysis (Fig. 1). The median (IQR) BG was 66 mg/dL (60, 68) with a range of 14–70 mg/dL. The population was middle-aged (median age 53 years), predominantly female (57.8%), and African American (62.2%). The most prevalent hypoglycemic risk factors were T2DM (36.2%), HIV (26.4%), insulin use (14.3%), CHF (14.3%), and CLD (10.7%). Other types of diabetes were less common: T1DM (1.5%), CF related diabetes (0.1%), and post-pancreatectomy diabetes (0.2%). Low-risk antihyperglycemic medications were prescribed in 8.3% of patients. There was a low prevalence of medications that have been rarely reported to cause hypoglycemia, such as indomethacin (0.1%), lithium (0.7%), and fluoroquinolones (1.2%).

**Table 2:**
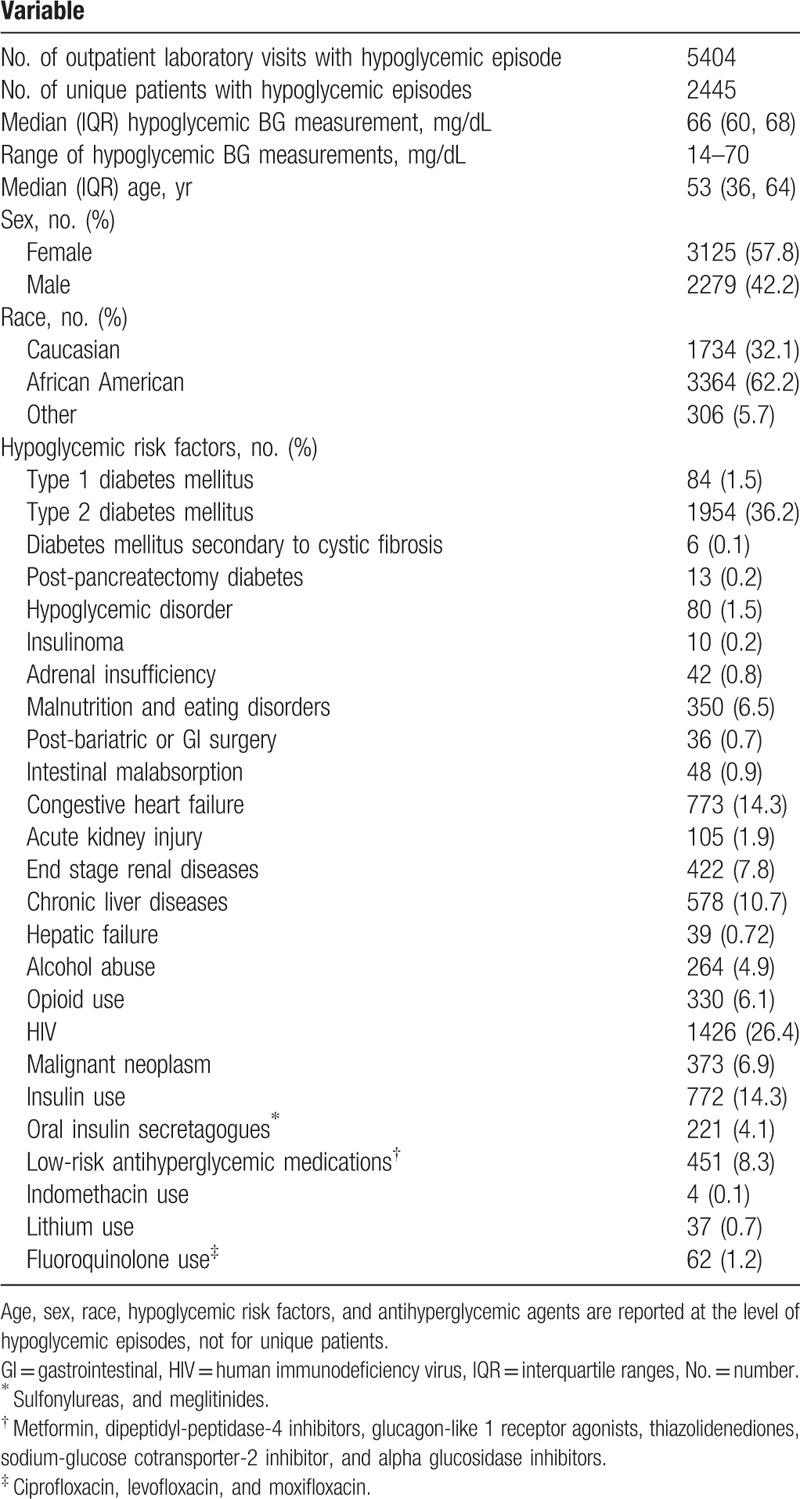
Characteristics of study population.

**Figure 2:**
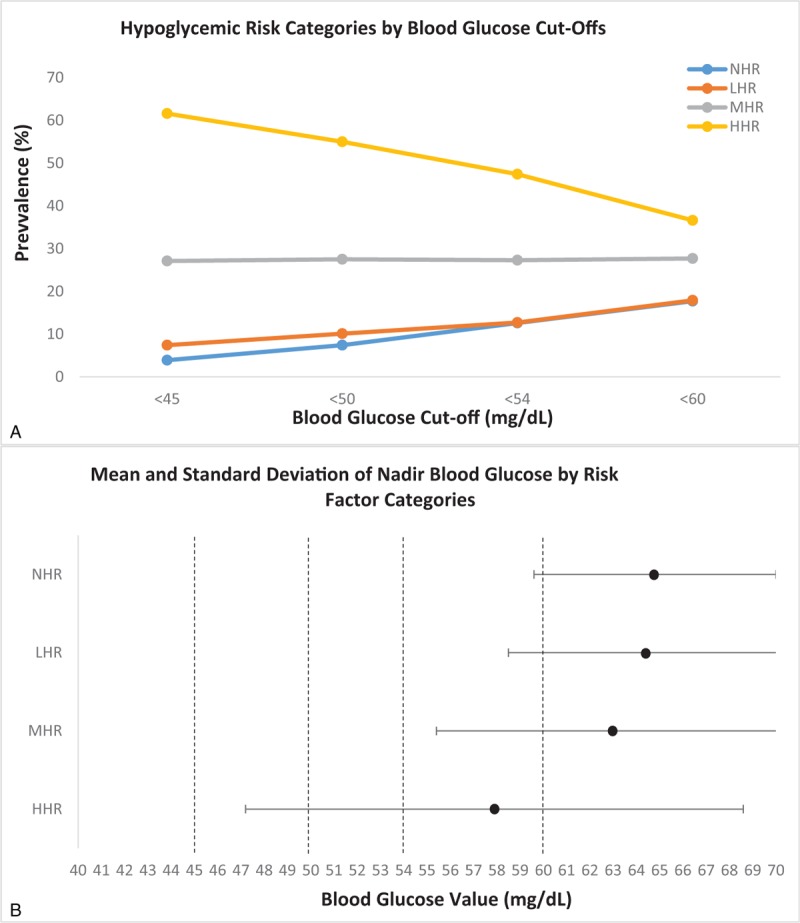
(A) Hypoglycemic risk categories by blood glucose cut-offs. (B) Mean and standard deviation of nadir blood glucose by risk factor categories. HHR = high hypoglycemic risk, LHR = low hypoglycemic risk, MHR = moderate hypoglycemic risk, NHR = no hypoglycemic risk.

**Figure 3:**
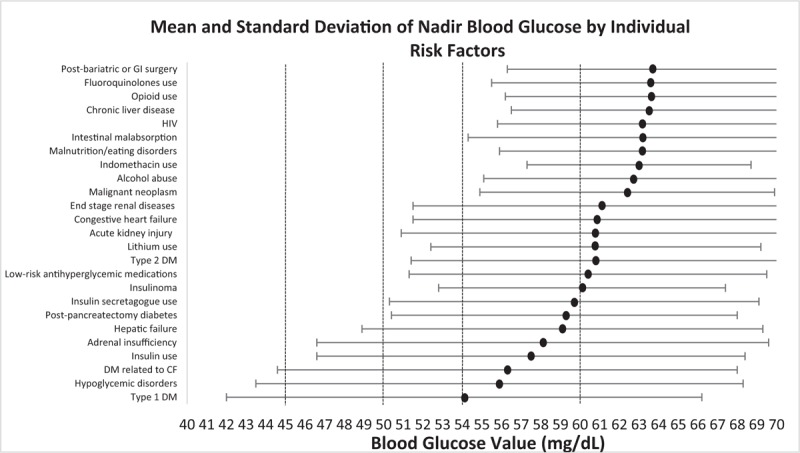
Mean and standard deviation of nadir blood glucose by individual risk factors. CF = cystic-fibrosis related diabetes mellitus, DM = diabetes mellitus, GI = gastrointestinal, HIV = human immunodeficiency virus.

Based on these HREs, the number of index BG events classified as NHR, LHR, MHR, and HHR were 1641 (30.3%), 1253 (23.2%), 1537 (28.5%), and 973 (18.0%), respectively. Figure 2A shows the prevalence of hypoglycemic risk categories by BG cut-offs. An inverse relationship between BG cut-off and HHR was observed, with the prevalence of HHR being 36.6% and 61.6% at BG cut-offs of <60 and <45 mg/dL, respectively. For the MHR group, there was a relatively stable prevalence across BG cut-offs. For LHR and NHR groups, there was a direct relationship noted. For example, the prevalence of LHR at <60 and <45 mg/dL was 17.2% and 6.9%, respectively. Interestingly, the mean ± standard deviation (SD) of BG was similar among the NHR and LHR groups (64.8 ± 5.3 and 64.5 ± 5.9 mg/dL, respectively; *P* = .58), slightly lower for the MHR group (63.1 ± 7.6 mg/dL; *P* = <.001 compared to NHR), and markedly lower for the HHR group (57.9 ± 10.7 mg/dL; *P* < .001 compared to NHR) (Fig. 2B). Figure 3 shows the mean ± SD BGV by individual hypoglycemic risk factors.

Table 3 shows the association of hypoglycemic BG thresholds and the hypoglycemic risk categories. In both univariate and fully adjusted models, there was an inverse association between BG thresholds and HHR. For example, the adjusted odds ratios of having a BG <60, <54, <50, and <45 mg/dL for the HHR group compared to the NHR group were 5.0 (95% confidence interval [CI] 3.9–6.3), 7.8 (95% CI 5.2–11.6), 13.6 (95% CI 8.0–23.3), and 27.3 (95% 11.5–64.3), respectively. Consistent with the relationship observed in Figure 2A, there was a less steep inverse relationship noted in the adjusted odds ratios by BG cut-offs in the MHR group, and relatively flat association for the LHR group.

**Table 3:**
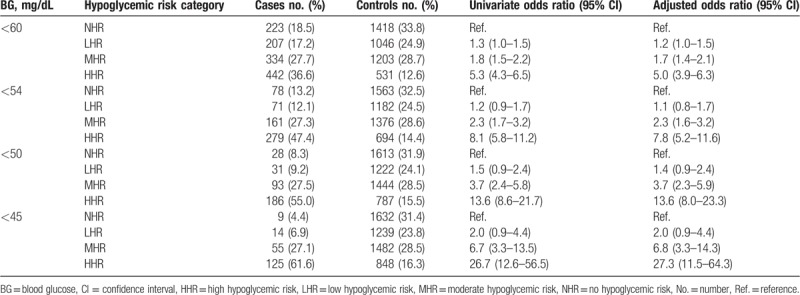
Unadjusted and adjusted associations of BG level and hypoglycemic risk categories.

Table 4 shows the test performance characteristics of the 4 BG cut-offs in classification of the hypoglycemic risk categories. For classification of MHR and LHR, all BG cut-offs had poor discrimination, with only 53–56% of cases being correctly classified compared to the NHR category. Although the specificity was relatively high, there was very low sensitivity in classification of MHR and LHR, with very high false-negative rates and low positive likelihood ratios. On the other hand, the BG cut-offs achieved better discrimination for classification of HHR, with 67.2% to 71.2% being correctly classified compared to the NHR control. Among the pre-selected BG cut-offs we evaluated, a BG <60 mg/dL had the highest accuracy in classification of HHR, but the positive likelihood ratio of 3.3 indicated only modest increase in the probability of HHR. As BG cut-offs decreased, there were substantial increases in the positive likelihood ratios, with BG <54, <50, <45 mg/dL having positive likelihood ratios (indicator in increase in posttest probability of disease) of 6.0 (moderate increase), 11.2 (large increase), and 23.4 (very large increase). There was an inverse relationship between the BG cut-off and specificity, positive likelihood ratios, and false-negative rates for classification of HHR. When comparing the BG cut-offs of <54 to <50 mg/dL, there was essentially no difference in the proportion of cases that were correctly classified (70.5% vs 68.8%), an increase in the false-negative rate (71.2%–80.9%) and a marked increase in the positive likelihood ratio (6.0–11.2).

**Table 4:**
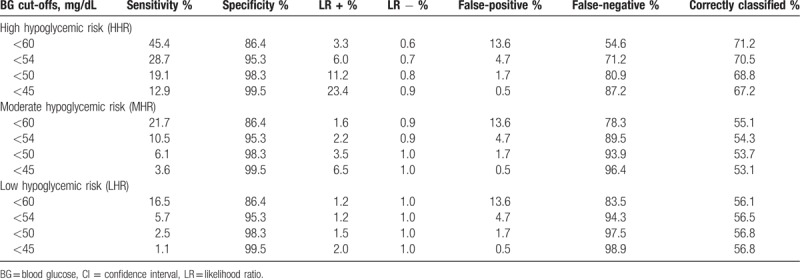
Test performance characteristics of four hypoglycemic BG thresholds in classification of hypoglycemic risk categories.

**Figure 4:**
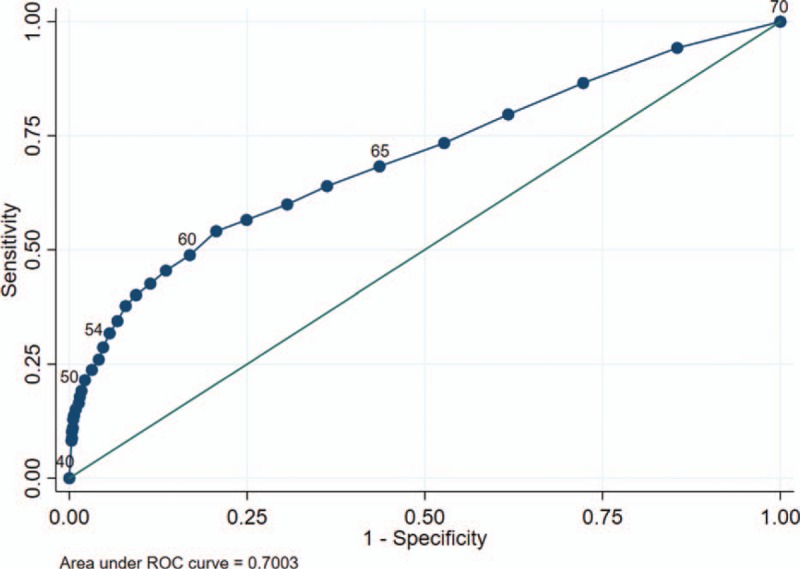
Receiver operating characteristic (ROC) curve of blood glucose levels for classification of high hypoglycemic risk.

The ROC curve for HHR classification with BG cut-offs decreasing by 1 mg/dL increments is shown in Figure 4. Of note, although the c-statistic is highest at a BG cut-off of 61 mg/dL, the positive likelihood ratio (an indicator of disease probability that is independent of disease prevalence) increases substantially with further declining BG values.

## 4 Discussion

In this study, we identified at least 1 hypoglycemic risk factor in nearly 70% of serum glucose results ≤70 mg/dL among general ambulatory patients. However, less than 20% of low BG results were obtained from patients with a condition considered to be high-risk for clinically significant hypoglycemia. While many clinical conditions and medications are associated with hypoglycemia, data from our population suggest that generally only high-risk clinical conditions predispose severe biochemical hypoglycemia. We noted an inverse relationship between BGV and the prevalence of HHR conditions, while a flat or direct relationship was observed with moderate and LHR conditions.

Although the prevalence of hypoglycemia in diabetic patients has been reported previously,^[5,35]^ the prevalence of hypoglycemia in general ambulatory patients has not been well studied. Specifically, the proportion of ambulatory patients with low BG (≤70 mg/dL), who have no identifiable hypoglycemic risk factors, is unknown. In our cohort, we found that 38% of hypoglycemic episodes occurred in patients with diabetes mellitus of any type, whereas 62% were nondiabetes related. Low BGV may occur in healthy patients in the setting of prolonged fasting, recent exercise, or reactive hypoglycemia due to recent meal intake.^[11]^ Since most nondiabetic individuals would be expected to have an intact counter-regulatory hormonal response to declining BG, it would be unusual for non-diabetic individuals to experience spontaneous BGVs <60 mg/dL in the absence of hypoglycemic symptoms. In healthy patients, autonomic symptoms typically occur at a BG threshold of <60 mg/dL and neuroglycopenic symptoms at a threshold <50 mg/dL.^[36]^

Thus, while it is reasonable to assume that healthy non-diabetic individuals might have mildly low BGVs above the counterregulatory thresholds (ie, BG 60–70 mg/dL), we found that 8.3%, and 4.4% of the NHR group (most likely to include non-diabetic healthy individuals) had BGV <50 mg/dL and <45 mg/dL, respectively. Low BGVs to this degree would not be expected in a healthy group of patients. Given the retrospective nature of this EMR-based study, we do not know whether this small subset of patients was symptomatic at the time of their lab draw. We suspect that their low BG readings were most likely due to missing information from the EMR, such as diagnostic codes and/or prescriptions for glucose-lowering medications resulting in misclassification as NHR, rather than laboratory error or some other factor.

As expected, we found a clear inverse relationship between the BGV and the odds of being in the HHR category, after adjusting for age, sex, and race. BG cut-offs were more accurate at classifying HHR than MHR and LHR. The proportion of cases correctly identified as HHR relative to the NHR control group was essentially the same across all BG cut-offs (67.2%–71.2%). Lowering the BG cut-off resulted in increasing specificity at the expense of lowered sensitivity. It appears that the BG cut-off of <54 mg/dL is the first point at which the positive likelihood ratio exceeds 5, a value that reflects a moderate (30%) increase in the probability of having the disease (in this case, HHR).^[37]^ Although a cut-off <60 mg/dL achieved the best classification accuracy, the positive likelihood ratio for HHR was only 3.3 at this cut-point, indicating only a slight (∼20%) increase in probability.^[37]^ Similarly, although a BG cut-off <45 mg/dL achieved the highest positive likelihood ratio for HHR (23.4), this cut-off achieved the lowest sensitivity (12.9%) and lowest classification accuracy (67.2%). Furthermore, a CAV BG cut-off this low would make it challenging for clinicians to contact patients in a reasonable time frame to intervene before the onset of hypoglycemic symptoms. Considering the fact that the positive likelihood ratio for HHR first exceeds 10 (indicator of high probability of disease) at the BG cut-off of <50 mg/dL, this may be a reasonable threshold to use as a CAV until further studies can evaluate the presence of patient-related adverse events or symptoms at specific BGVs in an ambulatory population.

In the absence of more precise models, our data suggest that BGV <54 mg/dL or <50 mg/dL are reasonable candidates for BG CAV. Raising the BG CAV cut-off from <50 mg/dL (our current CAV) to <54 mg/dL (consensus definition) would have the following implications: ∼3% increase in false-positive rate, ∼10% decrease in false-negative rate, and reduction from large increase to moderate increase in the probability of HHR based on the positive likelihood ratio. Since clinical outcomes or symptoms of patients were not available in this dataset, it is difficult to provide a specific recommendation for health systems in selecting a BG CAV. Further prospective studies that systematically collect information about HREs, signs, symptoms and other adverse events related to hypoglycemia (such as motor vehicle accident and ED visits) are needed for a complete risk-benefit analysis. Moreover, it is unknown how clinicians respond to CAV communications from clinical laboratories and how clinician behavior modifies patient behavior and/or outcomes. Whereas the inpatient setting may result in more standardized action by healthcare teams in response to a CAV for BG, it is likely that outpatient-based providers have variable responses. Further prospective studies that rigorously monitor clinician response to BG CAV are needed to determine whether this intervention is effective at preventing patient harm. In addition, individual institutions might need to analyze BG data with respect to their own unique patient populations to determine a meaningful CAV.

There are several strengths of this study. We used a large dataset from a general ambulatory population, so the findings were generalizable to most health systems. We included a comprehensive number of hypoglycemic risk factors, which we were able to validate as being present on or before the date of the BGV. The main limitation of this study was the assumption that HREs actually translates to clinically significant hypoglycemia. There are currently no validated hypoglycemic risk scores or classification methods that have been developed against a gold standard (ie, observed or patient-reported hypoglycemic symptoms). Thus, we were compelled to develop our own risk classifications based on clinical judgment and review of the literature. While medications were included as risk factors, doses or medication compliance could not easily be extracted from the EMR. We did not have information about body weight, which could be a potential hypoglycemic risk factor; however, we did include ICD-10 codes associated with underweight, weight loss, malnutrition, and eating disorders. Finally, although diabetes duration may be a risk factor for hypoglycemic unawareness, this information was not readily available. Inaccurate coding or underreporting of diagnoses also cannot be excluded.

## 5 Conclusions

In summary, while nearly 70% of ambulatory patients with BGV ≤70 mg/dL have at least 1 hypoglycemic risk factor, only ∼20% have exposure to high-risk medications or clinical conditions that could result in rapid deterioration in BGV. LHR and MHR factors do not correlate well with BG cut-offs in the hypoglycemic range. Although there is stronger correlation with BG cut-offs with HHR conditions, more information is needed regarding patient symptoms and impact of provider communication on clinical outcomes when selecting a CAV for BG to be applied to a general ambulatory population. In the meantime, BG CAVs of <54 or <50 mg/dL are both reasonable on the basis of their positive likelihood ratios, and selection of either would need to be balance perceived benefit from earlier identification against available costs and resources.

## Author contributions

**Conceptualization:** Mohammed S. Abusamaan, Mark A. Marzinke, Aditya Ashok, Karen C. Carroll, Kendall F. Moseley, Nestoras N. Mathioudakis.

**Data curation:** Mohammed S. Abusamaan, Nestoras N. Mathioudakis.

**Formal analysis:** Mohammed S. Abusamaan, Kathryn A. Carson, Nestoras N. Mathioudakis.

**Funding acquisition:** Nestoras N. Mathioudakis.

**Methodology:** Mohammed S. Abusamaan, Mark A. Marzinke, Nestoras N. Mathioudakis.

**Project administration:** Mohammed S. Abusamaan, Nestoras N. Mathioudakis.

**Supervision:** Nestoras N. Mathioudakis.

**Validation:** Mohammed S. Abusamaan, Nestoras N. Mathioudakis.

**Writing – original draft:** Mohammed S. Abusamaan, Nestoras N. Mathioudakis.

**Writing – review and editing:** Mohammed S. Abusamaan, Mark A. Marzinke, Aditya Ashok, Karen C. Carroll, Kyrstin Lane, Rebecca Jeun, Kendall F. Moseley, Kathryn A. Carson, Nestoras N. Mathioudakis.

Mohammed S. Abusamaan orcid: 0000-0001-9307-1315.

Mohammed S. Abusamaan orcid: 0000-0001-9307-1315.
